# Rubella seroprevalence among unvaccinated school-aged children in Jos, North Central, Nigeria

**DOI:** 10.11604/pamj.2024.49.1.44172

**Published:** 2024-09-02

**Authors:** Hyelshilni Samuel Waziri, Fatima Giwa, Adebola Tolulope Olayinka, Ndadilnasiya Endie Waziri, Clement Koeloengan Da'am, Yahaya Mohammed, Patrick Nguku, Samuel Abednego Dahal, Ugochukwu Nwokoro, Joseph Nakah, Yadang Dasohot Maktep, Ahmed Olowo-Okere

**Affiliations:** 1 African Field Epidemiology Network, Abuja, Nigeria,; 2 Ahmadu Bello University Zaria, Kaduna State, Nigeria,; 3 Africa Centres for Disease Control and Prevention, Jabi, Abuja, Nigeria,; 4 Department of Medical Microbiology, Jos University Teaching Hospital, Plateau State, Nigeria,; 5 Department of Community Medicine, University of Nigeria Teaching Hospital, Enugu State, Nigeria,; 6 Department of Pharmaceutical Microbiology and Biotechnology, Faculty of Pharmaceutical Sciences, University of Abuja, Abuja, Nigeria

**Keywords:** Rubella virus, seroprevalence, immunization, Nigeria

## Abstract

**Introduction:**

rubella poses a significant public health threat, particularly in developing countries, where congenital rubella remains a preventable concern. This cross-sectional study examined rubella seroprevalence among children aged 10 and under from May to September 2016 in Jos, Nigeria.

**Methods:**

using a multistage sampling method, eligible participants who had not been vaccinated against the rubella virus and consented to participate in the study were recruited across schools in the city. Rubella-specific IgG and IgM antibodies were detected from eluted serum collected from the participants using the enzyme-linked immunosorbent assay (ELISA). Data analysis and visualization was done using the R software version 4.3.1.

**Results:**

of the 405 participants investigated in this study, 336 (82.96%) tested positive for rubella IgG, while 9 (2.22%) tested positive for rubella IgM. Factors such as age ≥ 5 years and lack of Western education showed significant associations with rubella seropositivity.

**Conclusion:**

this study highlights the seroprevalence of rubella IgG and IgM antibodies among children aged 10 and under in Jos, Nigeria. The significant associations between rubella seropositivity and factors such as age ≥ 5 years and lack of Western education underscore the necessity for an effective rubella vaccination program to prevent congenital rubella syndrome (CRS).

## Introduction

Rubella also known as German measles, is a vaccine-preventable acute viral disease, characterized by mild fever and a distinctive maculopapular rash, which is similar in presentation to measles viral infection [1-3]. The disease is caused by the rubella virus (RV), the only member of the Rubivirus genus within the Togavirideae family [4]. This virus is spherical, with dimensions ranging from 50-85 nanometers, and possesses a positive-sense, single-stranded RNA genome [4,5]. Transmission typically occurs through droplets from the respiratory secretions of infected individuals, as well as through direct contact [4].

While a large proportion of rubella infections in children and adults are usually subclinical, contracting rubella during early pregnancy can lead to adverse outcomes [3]. Because of the teratogenicity of RV, neonates infected transplacentally usually experience congenital rubella syndrome (CRS), a range of congenital abnormalities characterized by premature delivery, low-birth weight, anaemia, thrombocytopenia, hepatitis, and serious encephalitis [3]. It may also present as blindness, deafness, heart defects, and developmental delays, among others [3]. A study has also linked CRS with autism [6]. The foetus of women infected with RV before or during the first trimester of pregnancy has up to 90% likelihood of developing CRS [7]. Other reports have suggested that women infected with RV during early pregnancy may suffer from miscarriages, spontaneous abortion, and stillbirth [2,3,8].

The global adoption of safe and efficacious live-attenuated vaccines has led to substantial reduction in the burden of RV infection [9]. Between 2007 and 2018, global annual incidence has reduced from 13.9 cases per million to 1.7 cases per million [10]. The number of reported rubella cases has further declined to 17,865 in 2022 [11]. In China, for example, the incidence of rubella has reduced from 9.11/100,000 in 2008 to 0.12/100,000 in 2017 [12]. Only 88 cases of CRS were registered and only 173 cases of rubella disease during pregnancy were reported in Italy between 2005 and February 2018 [2]. As many as 94 countries particularly the middle and high income have successfully eliminated rubella [11].

In Nigeria as well as other sub-Saharan Africa countries, the overall seroprevalence of rubella remains high [7,9,13], with studies indicating rates as high as 68% in certain regions [1]. However, there are variations in seroprevalence rates across different areas and populations within Nigeria [8,14,15]. For instance, among women with recurrent miscarriages who have never received the RV vaccine, seroprevalence rates of rubella IgG and IgM of 85% and 16.7% have been reported in Jos, north-central Nigeria [8]. Among pregnant women attending a tertiary healthcare facility in Kano, northwest Nigeria, an overall rubella seroprevalence was found to be 68.7% [14].

To effectively control and eventually eliminate rubella, it is crucial to understand the extent of immunity within different population groups to guide vaccination strategies and mitigate the risk of congenital rubella syndrome (CRS). This study specifically focused on the seroprevalence and factors associated with rubella-specific antibodies among school-aged children under 10 years old in Jos, north-central Nigeria. This age group represents a significant portion of the population that has not yet been fully reached by routine rubella vaccination programs, making it a key demographic for understanding baseline immunity levels. This project would provide insights into the burden of rubella infection, inform future vaccination strategies, and ultimately, it will contribute to the development of evidence-based policies and interventions aimed at reducing the burden of rubella and preventing the devastating consequences of CRS.

## Methods

**Study site:** the study focused on nursery and primary schools located within Jos metropolis, the capital city of Plateau State, Nigeria. The state is located between latitude 80°24'N and longitude 80°32' and 100°38' east and at an altitude of about 1,200 to 1,829 meters above sea level [16]. It is located approximately in the centre of the country and about 291 kilometres from Abuja, the Nigeria Federal Capital Territory [16]. It is bordered by Bauchi State to the Northeast, Kaduna State to the Northwest, Nasarawa State to the Southwest, and Taraba State to the Southeast. It has a landmass of about 21,000 sq. km and a projected population of 3,933,822 based on the 2006 National Population Census [16]. At the time of this study, there were 101 and 346 registered nursery and primary schools in Jos North and Jos South local government areas (LGA), respectively.

**Study design and population:** the study was a cross-sectional descriptive study carried out from May to September 2016. The study population consisted of pupils under 10 years of age and their caregivers from a total of 22 schools distributed across both Jos North and Jos South LGAs.

**Inclusion criteria:** all children who were 10 years of age and below attending the selected schools and who consented willingly to participate in the study.

**Exclusion criteria:** children aged 10 years and below who declined consent to participate in the study and those who had previously received the rubella vaccine were excluded.

**Sample size and sampling technique:** the sample size of 381 was calculated using the Thrushfield (1997) formula based on an expected prevalence rate of 45.2%, a desired precision of 5% at a 95% confidence interval, and a standard normal variate of 1.96 [15,17]. A two-stage modified cluster sampling method was employed, involving a random selection of schools from Jos North and Jos South, with proportional allocation based on the number of registered schools in the LGAs. Jos North has 101 registered schools, and Jos South has 346 registered schools. From the selected schools, an average of 18 pupils were sampled, with slight variations in some schools [18]. Participants were randomly selected from school registers until the sample size was achieved.

**Sample and data collection:** a pre-tested structured questionnaire was used to collect data on socio-demographics, social and past medical history of all participants. From participants who consented to participate in the study, 3ml of venous blood was collected under aseptic conditions. Blood was collected using sterile syringes and needles into plain red top venepuncture tubes, then stored in coolers with ice packs for transportation to the laboratory. After clotting, serum was separated from cells via centrifugation, collected using Pasteur pipettes into plain cryovials, and stored at -20°C for approximately 20 days. Standard laboratory procedure was strictly followed to ensure sample integrity, avoiding contamination and repetitive freezing and thawing.

**Sample processing:** rubella virus IgM/IgG was qualitatively detected in the collected samples using the prestige diagnostics® rubella IgM/IgG Kit following the manufacturer´s instructions. Briefly, after bringing all reagents and controls to room temperature, 100µl of sample diluent was added to appropriate wells followed by 10µl of the sample. Negative and positive controls were added accordingly. The microplate was then gently swirled and covered for a 20-minute incubation at 37°C. After incubation, wells were washed and HRP Conjugate was added. Following another 20-minute incubation, substrates A and B were added, incubated for 10 minutes, and then stop solution added. Absorbance was thereafter read at 450/630nm using a microplate reader. The cut-off optical density (OD) was determined as 2.1 times the negative control OD. If the negative control OD was below 0.09, a fixed absorbance of 0.09 was used for the cut-off calculation. The interpretation was based on comparing sample OD to the cut-off OD. The samples with OD greater than or equal to the cut-off were considered positive for rubella IgG/IgM, while those with OD below the cut-off were deemed negative.

**Data analysis:** it was conducted using the R software version 4.3.1. Descriptive statistics were employed for data analysis, and the association between positive rubella IgM and IgG samples and other risk factors was assessed through bivariate analysis. Statistical significance was set at a p-value of ≤ 0.05.

**Ethical considerations:** ethical clearance was obtained from the Research Ethics Committee of Jos University Teaching Hospital, and permission was sought from the Plateau State Ministries of Health and Education, and also from the Plateau State Universal Basic Education Board. Participation was voluntary, and individuals had the option to opt-out without any negative repercussions. Consent was obtained from caregivers, and assent was obtained from participating children. Confidentiality was strictly maintained throughout the study, and the data collected was solely used for this research.

## Results

This study involved a total of 405 children alongside their caregivers with a 100% response rate. The majority of children recruited for this study were aged between 7 and 8 years (27.90%), with a mean age of 6.33 years (SD ±2.523). Females constituted 54.32% of the children, most attended primary schools (70.02%), and the majority resided in Jos South (72.59%) and attended public schools (61.98%) (Table 1). The age of caregivers ranged from 20 to 49 years, mean age of 33.75 years (SD ±5.30), with the highest proportion falling in the 30-34 years age group (34.07%). The majority of participants were female (85.93%) and married (96.54%), predominantly in monogamous relationships (78.04%). Educational backgrounds varied, with 31.11% having no formal education and only 29.63% possessing tertiary education.

**Table 1:** socio-demographic characteristics of participants studied

Variable	Frequency	Proportion %
**Age of children (years)**		
1-2	43	10.62
3-4	59	14.57
5-6	95	23.46
7-8	113	27.9
9-10	95	23.46
**Sex of child**		
Male	185	45.68
Female	220	54.32
Type of school		
Nursery	119	29.38
Primary	286	70.62
**Type of school ownership**		
Private	154	38.02
Public	251	61.98
**Age of caregiver**		
20-24	5	1.23
25-29	88	21.73
30-34	138	34.07
35-39	111	27.71
40-44	50	12.35
≥45	13	3.21
**Sex of caregiver**		
Male	57	14.07
Female	348	85.93
**Marital status**		
Single	5	1.23
Married	391	96.54
Divorced	5	1.23
Widowed	4	0.99
**Type of marriage**		
Monogamy	313	78.04
Polygamy	85	21.96
**Level of education**		
None	126	31.11
Islamic	11	2.72
Primary	54	13.33
Secondary	94	23.21
Tertiary	120	29.63
**Occupation**		
Artisan	43	10.62
Banker	3	0.74
Civil servant	112	27.65
Farmer	11	2.72
Housewife	107	26.42
Security	3	0.74
Trader	126	31.11
**Place of residence**		
Jos North	111	27.41
Jos South	294	72.59

**Figure 1:**
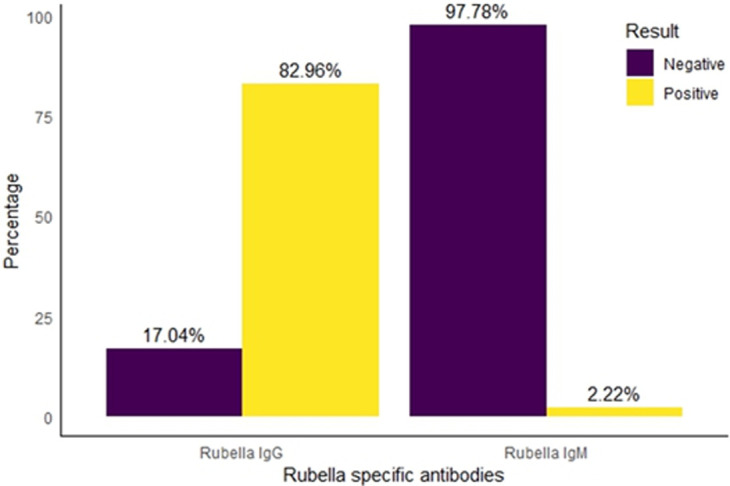
prevalence of rubella specific antibodies among the studied

**Rubella virus seropositivity:** overall, 336 (82.96%) tested positive for rubella IgG, while 69 (17.04%) tested negative. Additionally, only 9 (2.22%) of the children examined tested positive for rubella IgM antibodies (Figure 1).

**Risk factors for rubella virus antibodies seropositivity:** this result of associations between various factors and rubella IgG positivity is presented in Table 2. A higher proportion of IgG-positive cases was observed among children aged ≥5 years (258, 63.70%) compared to those <5 years (78, 19.26%). Similarly, a larger proportion of IgG-positive cases was found among children of caregivers with Western education (213, 52.59%) compared to those with no Western education (123, 30.37%). The chi-square test (χ^2^) of independence revealed significant associations between the educational level of caregivers (χ^2^= 6.10, p = 0.014) and the local government area (LGA) of residence (χ^2^= 20.85, p < 0.001) with IgG seropositivity. Other factors such as marital status, occupation of caregivers, and type of school ownership exhibited no significant relationship with IgG status (p > 0.05).

**Table 2:** factors associated with rubella virus infection among children and their caregivers

Variables	IgG Positive	IgG Negative	χ^2^	P-value
**Age**			3.475	0.0623
≥5 years	78	45		
<5 years	258	24		
**The educational level of the caregiver**			6.0996	0.0135
No western education	123	14		
Western education	213	55		
**Marital status**			0	1
Married	332	68		
Unmarried	4	1		
**Occupation of caregivers**			0	1
No gainful employment	89	18		
Gainful employment	247	51		
**Number of people in a room**			2.2664	0.1322
>3	192	32		
≤3	144	37		
**LGA of residence**			20.8533	0.0000
Jos North	108	3		
Jos South	228	66		
**Type of school ownership**			0.0051	0.9429
Private	127	27		
Public	209	42		
**Gender of child**			0.2764	0.5991
Male	151	34		
Female	185	35		
**Gender of caregiver**			0.0064	0.936
Male	48	9		
Female	288	60		
**Type of school**			1.5035	0.2201
Nursery	94	25		
Primary	242	44		
**Number of windows in the room where the child sleeps**			2.2413	0.1344
<2	117	17		
≥2	219	52		
**Cross-ventilation in the room child sleeps**			2.856	0.091
Yes	194	48		
No	142	21		
**Washing hands after wiping the child's nose**			0.1946	0.6591
Yes	187	41		
No	149	28		
**Sucking mucus from a child's nose**			0.827	0.3631
Yes	196	40		
No	140	29		
**History of fever in the last 3 weeks**			0.2315	0.6304
Yes	104	24		
No	232	45		
**History of rashes in the last 3 weeks**			1.8545	0.1733
Yes	32	11		
No	304	58		
**History of flu-like illness in the past 3 weeks**			0.4632	0.4961
Yes	86	21		
No	250	48		
**The rashes and flu-like illness present at the same time**			1.0135	0.3141
Yes	20	7		
No	316	62		
**Household member having flu-like illness in the past 3 weeks**			0.0227	0.8803
Yes	72	16		
No	264	53		
**History of blood transfusion**			1.6651	0.1969
Yes	25	9		
No	311	60		

Note: No Western education' here refers to individuals who pursue forms of education such as Arabic studies, which are distinct from the conventional Western education system and are highly prevalent in Northern Nigeria.

## Discussion

The global disparity in vaccine access poses a significant public health challenge, especially in developing nations. In Nigeria, despite various efforts such as national immunization campaigns and enhanced healthcare initiatives, to improve vaccination coverage, accessibility issues persist. Although the introduction of the rubella-containing vaccine (RCV) has been planned as part of the national routine immunization, it has not yet been implemented. Consequently, many school-aged children remain unvaccinated and susceptible to rubella. This study examines the seroprevalence of rubella virus (RV)-specific antibodies among unvaccinated school-aged children. Rubella-specific IgG serves as a long-term marker of prior infection, while IgM indicates recent infection.

The results revealed a high prevalence (82.96%) of rubella IgG among the studied children, which aligns with rates reported in other regions, such as Saudi Arabia and Kenya, where seropositivity rates of 90% and 80% respectively have been documented among school-aged children [19,20]. The high IgG seropositivity in these countries may be attributed to routine rubella vaccination. Consequently, a higher proportion of their children would have IgG antibodies against rubella. This is in contrast with our finding where high seropositivity was reported among cohorts of children who have not previously been immunized against RV. The high prevalence of rubella IgG among children who have not received the rubella vaccine suggests previous mild infections or natural exposure to the virus [21]. This highlights the need for epidemiological investigation to elucidate the underlying dynamics of rubella transmission in the study area. However, this prevalence sharply contrasts with the remarkably lower seropositivity of 1.8% observed in Tanzania among similar cohorts [4]. The difference in vaccination coverage, socio-economic factors, and healthcare infrastructure between Nigeria and Tanzania are likely responsible for a notable contrast in seropositivity rates [4]. Additionally, variations in the rate of natural rubella transmission within each community may also contribute to the observed differences.

This study observes a reduced prevalence of Rubella IgM (2.22%), a sharp contrast with a 2011 study in the study area which reported a significantly higher prevalence (45.2%) [16]. The notable decrease in RV IgM levels could potentially be attributed to the fact that rubella epidemics occur periodically usually between 3-5 years infections [22]. Additionally, differences in study cohorts could account for the large disparity between the current prevalence and the previous report, as the previous study only sampled hospitalized children [16]. The observed low IgM seropositivity rate is notably lower than the 23% previously reported in Uganda [23]. The finding of this study concurs with result of an epidemiological study of Pattern of Rubella in Africa which showed that RV IgM positivity is predominantly reported among children under 15 years of age [24].

The analysis of IgG seropositive children distribution revealed Local Government Area (LGA) of residence played a significant role, with residents of Jos South having a higher burden of seropositive children compared to Jos North. While both LGAs are urban, the increased likelihood in Jos South may stem from the crowded nature of its schools compared to Jos North, facilitating the rapid spread of rubella due to fewer schools in the former. This aligns with findings from a Tanzanian study linking rural residency to rubella seropositivity, highlighting the role of population density in disease transmission [4].

The higher rubella IgG positivity in children under 5 years (91.5%) compared to those of 5 years and older (63.4%), suggests earlier exposure to rubella among younger children. The observed pattern aligns with other studies in similar settings, which have shown that rubella transmission predominantly affects younger age groups, often before school age [7]. This may be due to the virus's high transmissibility in densely populated communities and the waning of maternal antibodies [25]. These findings highlight the importance of introducing rubella vaccination to protect all age groups, especially those who miss early exposure.

Given the significant number of individuals testing negative for rubella IgG antibodies (17.04%), there remains a considerable population susceptible to rubella. Thus, the Nigerian government must prioritize the introduction and routine administration of rubella vaccines, as recommended by the WHO, to prevent the spread of rubella and achieve a coverage level of 80% or greater [26]. Furthermore, comprehensive awareness campaigns are essential to educate the public about rubella and the associated risks of CRS. To ensure effective dissemination of information, resources such as educational materials should cover both rubella and CRS. Additionally, fostering collaboration and information sharing among key stakeholders, including epidemiologists, healthcare providers, and policymakers, is vital for implementing targeted strategies to combat rubella and mitigate its adverse effects.

**Study limitation:** while the study's methodology is robust, its focus on children under 10 years old and the urban setting of Jos may limit the generalizability of the findings. The study does not account for rubella seropositivity among older children, adults, or rural populations, which is critical for a comprehensive understanding of rubella immunity and CRS risk.

## Conclusion

This study reports a high seroprevalence of RV-specific antibodies among unvaccinated school-aged children in northcentral Nigeria indicating substantial natural immunity. However, a considerable proportion of the population (about 17%) remains susceptible to rubella. These findings underscore the urgent need for introduction of rubella-containing vaccines (RCV) in line with WHO targets to prevent future outbreaks and reduce the burden of rubella and its complications.

### What is known about this topic

- *Rubella is a vaccine-preventable disease caused by the rubella virus (RV), characterized by mild fever and a maculopapular rash, with severe consequences if contracted during early pregnancy, potentially leading to congenital rubella syndrome (CRS);*
- *Global adoption of rubella vaccination has significantly reduced the incidence of rubella and CRS, with some countries successfully eradicating the disease;*
- *In Nigeria, the seroprevalence of rubella remains high, with significant variations across different regions and populations*.

### What this study adds

- *This study provides updated data on rubella seroprevalence among unvaccinated school-aged children in Jos, North Central Nigeria, revealing that 82.96% tested positive for rubella IgG and 2.22% for rubella IgM;*
- *It identifies factors significantly associated with rubella seropositivity, such as age (≥ 5 years) and lack of Western education among caregivers;*
- *The findings underscore the need for an effective rubella vaccination program in high-risk regions to prevent CRS and reduce the overall burden of rubella infection*.
